# P-1161. Bacterial Bloodstream Infections in Pediatric Solid Organ Transplant Recipients within One Year of Transplant

**DOI:** 10.1093/ofid/ofae631.1347

**Published:** 2025-01-29

**Authors:** Mario M Landa, Ayelet Rosenthal, Caitlin Li, Mehreen Arshad, Sameer Patel, Larry K Kociolek, Alyah Barnes, William J Muller

**Affiliations:** Ann & Robert H. Lurie Children's Hospital of Chicago, Chicago, Illinois; Ann & Robert H. Lurie Children's Hospital of Chicago, Chicago, Illinois; Lurie Childrens Hospital, Chicago, Illinois; Northwestern University/Lurie Children's Hospital of Chicago, Chicago, IL; Ann and Robert H. Lurie Children's Hospital, Chicago, Illinois; Ann & Robert H. Lurie Children's Hospital of Chicago, Chicago, Illinois; Ann & Robert H. Lurie Children's Hospital of Chicago, Chicago, Illinois; Ann and Robert H. Lurie Children’s Hospital of Chicago and Northwestern University Feinberg School of Medicine, Chicago, Illinois

## Abstract

**Background:**

Bacterial bloodstream infections (BSI) are a major cause of morbidity in immunosuppressed children, yet data are limited for pediatric solid organ transplant recipients (SOT). Prior studies have found varying rates of BSI in the first 2 years after pediatric transplant. This single center study investigated the incidence, risk factors and outcomes of pediatric SOT recipients who experienced a BSI within 1 year of transplantation.

**Table 1.**
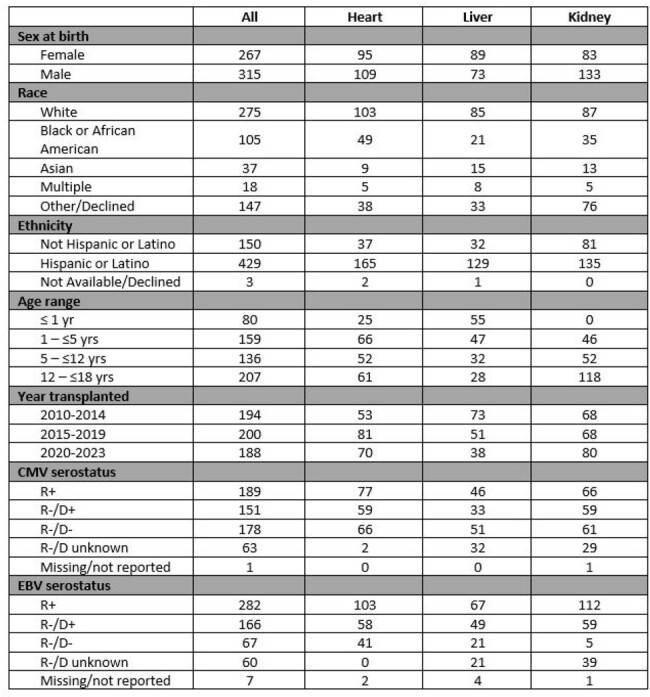
Patient demographics

**Methods:**

Retrospective single-center study of pediatric heart, liver, or kidney transplant recipients undergoing their first transplant at Lurie Children’s between January 2010 and December 2023. The primary outcome measure was bacterial bloodstream infection (BSI) within one year of SOT (minimum follow-up three months).

**Table 2.**
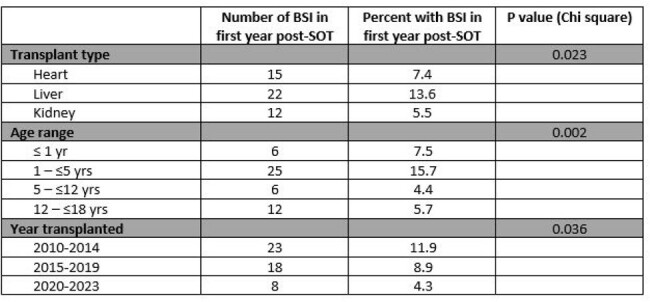
Results for primary outcome

**Results:**

A total of 582 patients under age 18 received a first SOT between January 1, 2010 and December 31, 2023 (Table 1). Overall incidence of BSI within one year of SOT was 8.6%. Rates were significantly higher in liver recipients (13.6%) than heart (7.4%) and kidney recipients (5.5%; P=0.02). Incidence was highest in patients transplanted between 1 and 5 years of age (15.7%) than in other age ranges (Table 2; P= 0.02). Among those with BSI, the median time to first episode after transplant was 20.5 days (IQR 11.3, 98.5; Table 3). The most common identified infectious source was a central venous catheter (Table 4), with 39 of the 49 episodes occurring while a central venous catheter was in place. The most common organisms identified were *Klebsiella pneumoniae* (8 patients), *Staphylococcus epidermidis* (7 patients), and *Enterobacter cloacae* (6 patients, 2 of whom had polymicrobial bacteremia). Overall incidence dropped significantly over the time frame analyzed, comparing those transplanted between 2010-14 (11.9%), between 2015-19 (8.9%), and 2020-2023 (4.3%); P=0.02.

**Table 3.**
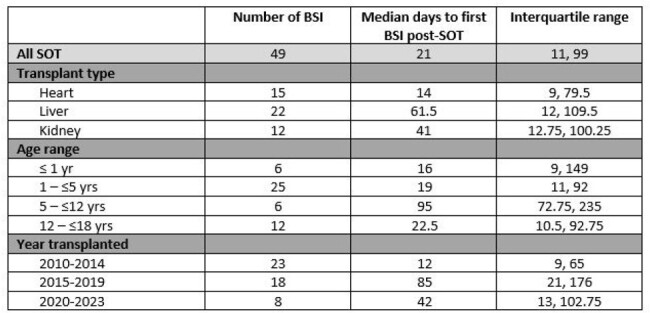
Median time to first BSI after SOT

**Conclusion:**

BSI within the first year after SOT is common in children, occurring at rates comparable to published data in adults. Children under 5 years of age had the highest rate, and presence of a central venous catheter was common during initial episodes. Further investigation is needed to identify more detailed risk factors associated with BSI in children receiving solid organ transplant, prevention strategies, and reasons contributing to the drop is BSI rates over time.

**Table 4.**
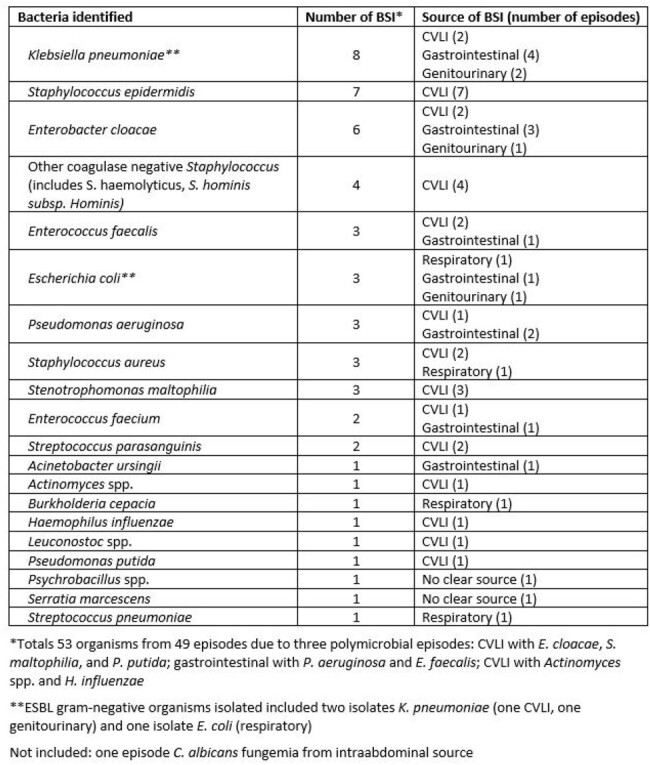
Organisms identified and source of infection

**Disclosures:**

**Larry K. Kociolek, MD, MSCI**, Merck: Grant/Research Support **William J. Muller, MD**, Ansun Biopharma: Grant/Research Support|Astellas Pharma: Advisor/Consultant|Astellas Pharma: Grant/Research Support|AstraZeneca: Advisor/Consultant|AstraZeneca: Grant/Research Support|DiaSorin: Advisor/Consultant|DiaSorin: Honoraria|Eli Lilly and Company: Grant/Research Support|Enanta Pharmaceuticals: Advisor/Consultant|Enanta Pharmaceuticals: Grant/Research Support|F. Hoffmann-LaRoche Ltd (Roche): Grant/Research Support|Finley Law Firm, P.C.: Advisor/Consultant|Gilead Sciences: Grant/Research Support|Melinta Therapeutics, Inc.: Grant/Research Support|Merck Sharpe & Dohme: Grant/Research Support|Moderna: Grant/Research Support|Nabriva Therapeutics, plc: Grant/Research Support|Paratek Pharmaceuticals, Inc.: Grant/Research Support|Pfizer: Grant/Research Support|ProventionBio: Advisor/Consultant|Sanofi: Advisor/Consultant|Sanofi: Honoraria|Tetraphase Pharmaceuticals, Inc.: Grant/Research Support

